# Supplementary material for: WordCommentsAnalyzer: A windows software tool for qualitative research
Source: F1000Res. 2018 Sep 4;7:536. Originally published 2018 May 3. [Version 2] doi: 10.12688/f1000research.14819.2 (PMC6137411; doi:10.12688/f1000research.14819.2)
Supplement: Supplementary file 3 [file f1000research-7-17614-s0002.tgz › b2a79496-4ada-47c4-9682-a99ce867836d.zip!/Eight Iranian Health Journals/Archives of Rehabilitation_64.docx]

© Archives of Rehabilitation. This work is licensed under CC BY-NC 4.0 (https://creativecommons.org/licenses/by-nc/4.0/).

Volume & Issue: دوره 4 - شماره 2

Title: مقایسه تأثیرات سه روش تمرین‌درمانی بر شدت درد و درصد ناتوانی افراد مبتلا به کمردرد مکانیکی مزمن

http://rehabilitationj.uswr.ac.ir/article-1-603-fa.html

هدف: هدف از طرح و اجرای این تحقیق ارزیابی سه روش متداول ورزش درومانی در بیماران مبتلا به کمر درد و مطالعه چگونگی تأثیر هر یک از این ورزش ها بر شدت درد و درصد ناتوانی ناشی از کمر درد است . روش بررسی: این مطالعه به صورت کارآزمایی بالینی بر روی 25 بیمار مبتلا به کمر درد مزمن مکانیکی با شرایط شغلی یکسان انجام شد. میزان شدت درد توسط جدول ارزیابی عددی درد ، و درصد ناتوانی ناشی از کمر درد توسط پرسشنامه اوسوستری مورد ارزیابی قرار گرفت. میزان اعتبار داده ها نیز بوسیله آزمون کندال محاسبه شد . افراد مورد تحقیق به سه زیر گروه تقسیم شدند به گونه ای که میانگین متغیرها در سه گروه ، اختلاف معنی داری با یکدیگر نداشتند . گروه اول با تعداد 8 نفر ورزش های ویلیامز را انجام دادند ، و گروه دوم با تعداد 7 نفر ورزش های مکنزی ، و گروه سوم با تعداد 10 نفر بودند که ورزش های ثبات دهنده را انجام دادند . طبق برنامه هر گروه طی دو مرحله 6 هفته ای ( 6 هفته اول 6 روز در هفته و 6 هفته دوم 3 روز در هفته – ( یکروز در میان ) و در مجموع 12 هفته ورزش های مربوط به خود را انجام دادند . ارزیابی آماری با استفاده از نرم افزار SPSS و انجام آزمون های ویلکاکسون – فریدمن – کروسکال والیس و من ویتنی انجام شد . یافته ها: نتایج به دست آمده نشان دادند که ورزش های انجام شده در هر سه گروه با کاربرد ورزش های تقویتی عضلات ستون فقرات می توانند برکاهش درد بیماران مؤثر باشند .البته زمان و سرعت کاهش درد، درصد ناتوانی و ماندگاری تأثیر ضد درد در گروهی که ورزش های ثبات دهنده را انجام داد با دوگروه دیگر اختلاف معنی دار وجود داشت. (0.01=P ) نتیجه گیری: بنابراین در هر سه روش درمانی درد بیمار کاهش می یابد ، لیکن ورزش های ویلیامز و مکنزی همیشه در شرایط استاتیک انجام می شوند و یک گروه عضلانی خاص را تقویت می کنند، اما در ورزش های ثبات دهنده ، علاوه بر تقویت عضلات در شرایط استاتیک تقویت عضلات، در شرایط دینامیک نیز صورت می گیرد . بیمار از شرایط انجام تمرینات سبک به سمت تمرینات سنگین، و در نهایت به سوی بهبود شرایط تعادل از دست رفته پیش می رود ، به همین دلیل انجام این ورزش ها علاوه بر تسریع در کاهش درد موجب بهبود شرایط ذهنی فرد، بهبود اعتماد به نفس و در نهایت کاهش درصد ناتوانی ناشی از کمر درد می شوند.

Title: همراهی الل T پلی‌مورفیسم جایگاه ۶۷ – پروموتر ژن انتقال دهنده دوپامین ( DAT1 ) با اسکیزوفرنیا

http://rehabilitationj.uswr.ac.ir/article-1-605-fa.html

هدف: نقص در سامانه ی نقل و انتقال دوپامین، می تواند به عنوان یکی از علل بیماری اسکیزوفرنی مطرح شود. ترانسپورتر دوپامین ( DAT1 ) باز جذب دوپامین از انتهای سیناپس های عصبی را بر عهده دارد، بنابراین نقش مهمی را در تنظیم نقل و انتقال دوپامین ایفا می کند. در این تحقیق ، همراهی احتمالی پلی مورفیسم 67T/A-   واقع در پروموترژن DAT1 با بیماری اسکیزوفرنیا به وسیله روش توصیفی مورد بررسی قرار می گیرد. روش بررسی:  یکصد بیمار مبتلا به اسکیزوفرنیا (طبق معیارهای DSM_1V) برحسب مصاحبه بالینی، و یکصد نفر گروه شاهد متناسب و غیر بیمار از نظر ژنوتیپ ها و درصد الل های مربوط به پلی مورفیسم مذکور بررسی شدند. یافته ها: درصد ژنوتیپ ها در گروه بیمار و شاهد به ترتیب ، به قرار زیر است: %12=TT ،   %59=AT   ،  %29=AA %5=TT   ،   %38=AT   ،  %57=AA نتیجه گیری: ارزشP   به دست آمده در تحقیق کمتر از 0003/0 می باشد. این یافته ها برای نخستین بار در جهان مدرکی دال بر ارتباط پلی مورفیسم واقع در پروموترژن DAT1، با سبب شناسی اختلال اسکیزوفرنیا در جمعیت مورد بررسی را نشان می دهد. برای ارزیابی بیشتر اهمیت یافته های ما پژوهشهای بیشتری با نمونه های مشتقل و بررسی های همراهی، بر پایه خانواده ضروری است.

Title: نشانگان روانشناختی اثر ضربه در کودکان آزاردیده

http://rehabilitationj.uswr.ac.ir/article-1-606-fa.html

هدف: حوادث آسیب زای بسیاری از جمله بلایای طبیعی، آزارهای جسمی، روان شناختی و جنسی برای کودکان حادث می شود. شواهد زیادی وجود دارد، مبنی براین که چنین تجاربی می توانند به مجموعه ای از آثار روان شناختی منتهی شوند. رویارویی کودکان با این ضربات دارای عوارض منفی بر سلامت روانی آنان است، اضطراب، افسردگی، فشار روانی پس از ضربه و گسستگی، خشم و پرخاشگری از جمله این عوارض است. به نظر می رسد که آثار حوادث آسیب زا به صورت معنا داری، به نوع و شدت ضربه مربوط باشد. هدف پژوهش حاضر بررسی رابطه بین شدت و نوع ضربه با اثرات ناشی از آنها است. روش بررسی: 3042 دانش آموز مذکر و مؤنث مدارس راهنمایی که در یک مطالعه زمینه یابی برای برآورد شیوع نقطه ای کودک آزاری شرکت کرده بودند ، فهرست نشانگان ضربه روانی-فرم الف (TSCC-A) و مقیاس خودگزارشی کودک آزاری (CASRS) را تکمیل کردند. پس از شناسایی کودکان آزار دیده، نشانگان ضربه در آنها مورد مقایسه قرار گرفت. TSCC-A سنجه ای خودگزارشی در مورد آشفتگی پس از ضربه و نشانه شناسی روان شناختی در کودکان 8 تا 16 ساله مذکر و مؤنث است. این سنجه در ارزیابی کودکانی که حوادث فشار از جمله حملات جسمی و جنسی ، قربانی شدن توسط هم سالان ، فقدان های اساسی ، مشاهده خشونت در دیگران و بلایای طبیعی را تجربه کرده اند مفید است. TSCC-A اشاره ای به موضوعات جنسی نمی کند. CASRS یک مقیاس خودگزارشی است که کودک آزاری و غفلت را با 38 ماده و چهار خرده مقیاس (آزار روان شناختی، غفلت، آزارجسمی و جنسی) می سنجد. یافته ها : با در نظر گرفتن نوع تجربه آسیب زا، نتایج مطالعه حاضر حاکی از آن است که کودکان آزار دیده در مقایسه با گروه آزار ندیده بطور قابل توجهی نمرات بالاتری را در مقیاس ها و خرده مقیاس های TSCC-A دریافت می نمایند. آنها خصوصاً نشانگان ضربه بیشتری را مقیاس افسردگی، اضطراب، فشار روانی پس از ضربه، خشم و گسستگی در مقایسه با کودکان بهنجار گزارش می کنند. نتیجه گیری: شدت نشانه شناسی حوادث آسیب زا به نوع و میزان این حوادث مربوط می باشد.

Title: بررسی مقایسه‌ای تأثیر خستگی عضلات پروگزیمال و دیستال اندام تحتانی بر کنترل پاسچرال مردان سالم

http://rehabilitationj.uswr.ac.ir/article-1-608-fa.html

هدف: مطالعات متعددی تأثیرات خستگی عضلانی، بر حس عمقی و کنترل عصبی عضلانی را نشان داده اند. با این حال، تمامی مطالعات موجود صرفاً تأثیر خستگی موضعی در عضلات ناحیه مچ پا، بر کنترل پاسچرال را مورد بررسی قرار داده اند و، تاکنون پژوهشی که به بررسی تأثیر خستگی عضلات پروگزیمال اندام تحتانی بر کنترل پاسچرال پرداخته باشد مشاهده نشده است. مقایسه میزان تغییرات در پارامترهای کنترل پاسچرال بعد از خستگی ایزوکینتیک عضلات پروگزیمال و دیستال اندام تحتانی. روش بررسی: نمونه مورد مطالعه تعداد 20 نفر مرد جوان سام بود (سن:4/2-/+6/22 سال، قد 6/3-/+7/173 سانتی متر، وزن: 9/7-/+3/63 کیلوگرم ). تعداد جلسه های آزمون 4 جلسه بود که ترتیب آنها براساس محل و صفحه خستگی به صورت تصادفی انتخاب می شد. با استفاده از دینامومتر ایزوکینتیک بایودکس، در هر یک از جلسات، خستگی در یکی از گروه های عضلانی زیر ایجاد می شد: پلانتار/دورسی فلکسورهای مچ پا، اورتور/اینوتورهای مچ پا، فلکسور/اکستنسورهای ران، و ابداکتور/بایودکس استفاده شد و شاخص های ثباتی کلی، قدامی /خلفی، و طرفی ثبت می شد. مقادیر بالاتر این شاخصها نشانگر مهارت تعادلی کمتری بود. یافته ها: تحلیل نتایج تعادلی قبل و بعد از خستگی در تمامی جلسه های آزمون، نشانگر افزایش معنی دار تمامی شاخص های ثباتی می باشد. تحلیل واریانس برای اندازه های مکرر بر روی میزان تغییرات شاخصهای ثباتی در هر جلسه آزمون صورت گرفت، نتایج نشان داد که خستگی عضلات مفصل ران باعث افزایش بیشتری در شاخصهای ثباتی نسبت به عضلات مچ پا می شود. نتیجه گیری: خستگی ایزوکنیتیک عضلات مچ و ران، باعث کاهش معنی دار توانایی کنترل پاسچرال در مردان سالم جوان می‌شود. به علاوه، یافته های تحقیق حاکی از آن است که عضلات مفصل ران نقش برجسته تری در کنترل پاسچرال دارند.

Title: بررسی سلامت روانی زنان سرپرست خانوار

http://rehabilitationj.uswr.ac.ir/article-1-609-fa.html

هدف: پژوهش حاضر به بررسی وضعیت سلامت روانی زنان سرپرست خانوار تحت پوشش سازمان بهزیستی استان تهران پرداخته است. سؤال اساسی مورد آزمایش این بود که آیا میزان سلامت روانی و علائم نه گانه آن برحسب آزمون SCL-90-R در زنان سرپرست خانوار تحت پوشش و غیر تحت پوشش سازمان مذکور متفاوت است. روش بررسی: 180 نفر زنان سرپرست خانوار تحت پوشش سازمان بهزیستی استان تهران به صورت تصادفی-سهمیه ای از بین زنان تحت پوشش انتخاب شدند. ابزار پژوهش عبارت از آزمون SCL-90-R و یک پرسشنامه اطلاعاتی، روش پژوهش مورد – شاهدی از نوع مقطعی بوده است. برای تجزیه و تحلیل داده ها از آزمون t تک نمونه ای، t مستقل، رگرسیون چند متغیری و آزمون فریدمن استفاده شده است. یافته ها: یافته های پژوهش نشان داد که میانگین حاصل از نمرات آزمون سلامت روانی SCL-90-R و علائم نه گانه آن در آزمودنی های گروه مورد به طور معنی داری بالاتر از میانگین حاصل از نمرات همان آزمون در جامعه بهنجار ایرانی است و حاکی از آن است که این زنان در معرض آسیب های روانی قرار دارند. نتیجه گیری: شدیدترین علائم در آزمودنی های گروه مورد افسردگی و شکایت جسمانی است، آزمودنی های گروه شاهد به طور معنی داری (P=0.016) ازآزمودنی های گروه مورد نیز افسرده ترند . همچنین درآمد زنان سرپرست خانوار، درآمد سرانه افراد تحت تکفل ایشان و سلامت جسمانی خود آنان نیز پیش بینی کننده های معتبری برای سلامت روانی این زنان می باشد و که این نتیجه مؤید یافته های پژوهشی های مربوط پیشین و مبین آن است که توجه به وضعیت اقتصادی و معیشتی این زنان حائز اهمیت خاص است.

Title: پیش‌بینی خطر زمین خوردن سالمندان آسایشگاهی با استفاده از دو روش ارزیابی عملکردی

http://rehabilitationj.uswr.ac.ir/article-1-610-fa.html

هدف: با افزایش سن در سالمندان توانایی هایشان کاهش می یابد ، و این کاهش توانایی ها باعث افزایش احتمال زمین خوردن و به تبع آن کاهش کیفیت زندگی فرد می شود. هدف این مطالعه شناسایی سالمندان مقیم در آسایشگاه می باشد که در معرض خطر زمین خوردن برای دستیابی به این هدف از دو روش ارزیابی عملکردی مقیاس تعادل برگ(1) و مقیاس تینه تی(2) استفاده شده و شاخص های اعتبار بهترین روش ، محاسبه گردید . روش بررسی: این طرح یک تحقیق متدولوژیک غیر مداخله ای آینده نگر ، از نوع مطالعه اعتبار افتراقی(3) است و برای ارزیابی مقایسه ای اعتبار افتراقی دو روش ارزیابی بالینی در سالمندان با و بدون سابقه زمین خوردن انجام شده است. تعداد زمین خوردن سالمندان آسایشگاهی در مدت 6 ماه به صورت آینده نگر بررسی شد . در پایان ، سالمندانی که در این مدت دوبار یا بیشتر زمین خورده بودند در گروه با سابقه زمین خوردن(4) و آنهایی که در این مدت زمین نخورده بودند در گروه بدون سابقه زمین خوردن(5) قرار داده شدند. نمونه ها شامل 21زن و 33 مرد با میانگین سنی 79-75 سال بودند ، ( انحراف معیار : 47/8 و دامنه 98-61 ) که در فعالیت های روزمره مستقل بوده و قادر به ده متر راه رفتن با یا بدون استفاده از وسایل کمکی و ... بودند و به طور آگاهانه داوطلب شرکت در مطالعه شدند. متغیرهای زمینه ای تحقیق عبارت بود از : سن ، جنس ، استفاده یا عدم استفاده از وسایل کمکی ، قد ، وزن ، تعداد داروهای مصرفی و تعداد بیماری . متغیر مستقل اصلی زمین خوردن و متغیر وابسته نتایج دو روش ارزیابی عملکردی بودند . یافته ها: آزمون  T مستقل نشان دهنده اختلاف آماری معنی دار میانگین امتیازهای مقیاس تعادل برگ (0.0001=P) و مقیاس تینه تی (0.0001=P ) در دو گروه با سابقه زمین خوردن و بدون سابقه زمین خوردن بود. نتیجه گیری: نتایج آزمون Logistic Regression نشان دهنده اعتبار افتراقی بسیار بالاتر نتایج آزمون مقیاس بالانس برگ، نسبت به مقیاس تینه تی بود. بررسی شاخص های اعتبار روش ارزیابی مقیاس تعادل برگ نشان می دهد که توجه به همه شاخص های اعتبار بایستی اساس تصمیم گیری های کلینیکی باشد.

Title: هنجاریابی آزمون لب‌خوانی سارا ( شماره ۲) بر روی بزرگسالان شنوا

http://rehabilitationj.uswr.ac.ir/article-1-611-fa.html

آزمون لب خوانی برای ارزیابی میزان توانایی لب خوانی و سطح بندی در آموزش لب خوانی به کار می روند ، و بر دو نوع اند تحلیلی و ترکیبی . آزمون شماره 2 سارا آزمونی تحلیلی و مکمل آزمون تکمیلی شماره 1 سارا (1) است. هدف:  ساخت آزمون لب خوانی ( واج های بی معنا ) و اجرای آن روی گروهی از افراد شنوا روش بررسی: پژوهش به شیوه توصیفی تحلیلی ، در دو مرحله تهیه مواد و اجرای آزمایشی انجام شده است. همه واج های زبان فارسی در زمینه واکه – همخوان – واکه قرار داده شدند. در مرحله اجرا 88 نفر با شنوایی و بینایی هنجار ( 58 مرد و 30 زن ) در محدوده سنی 85-17 سال مورد بررسی قرار گرفتند. یافته ها: 1- آزمون لب خوانی سارا ( شماره2 ) شامل 23 ماده آزمونی دوهجایی بی معنا . 2- توانایی تشخیص واجهای زبان فارسی در 6 گروه هم آوا . 3- روایی ، محتوایی آزمون 4- عدم وجود تفاوت معنادار بین نتایج آزمون و آزمون مجدد ( پایانی آزمون ). نتیجه گیری: در این پژوهش، آزمون لب خوانی شماره 2 سارا طراحی شد که آزمونی روا و پایا جهت ارزیابی توانایی لب خوانی واجهای زبان فارسی است . آزمون حاضر صرف نظر از بافت ساده اش، اطلاعات بسیار مفیدی در مورد توانایی لب خوانی افراد به ما ارائه می دهد و تکمیل کننده آزمون های ترکیبی ( با مواد آزمونی واژه با جمله ) است.

Title: تجویز و مصرف داروهای روان‌درمان بخش در کهنسالی

http://rehabilitationj.uswr.ac.ir/article-1-618-fa.html

درصد زیادی از افراد کهنسال، مبتلا به اختلالات روانی هستند و به طور معمول روان درمانی دارویی به منظور درمان و کنترل اختلالات رفتاری آنها مورد استفاده قرار می گیرد. کهنسالی، در بیشتر موارد به بیماری های مزمن و متعددی منجر می شود که موجب ناتوانی، کاهش عملکرد و ضعف عمومی در این افراد می شود. روند طبیعی پیری، خود به تنهایی بر لزوم تغییر در شیوه دارو درمانی را در افراد مسن تأکید می کند. بدلیل بروز بیماری های مختلف ناشی از افزایش سن الگوی مصرف دارو و تجویز داروهای متعدد، بخصوص در بیمارستان ها و خانه های سالمندان تغییر می کند. گوناگونی و تحولات معمول در شیوه زندگی افراد کهنسال نیز تأثیر عمده ای بر روش مصرف دارو در آنان دارد. این مقاله ضمن بررسی مقالات و منابع متعدد به بحث در مواردی چون مروری بر متابولیسم دارویی در افراد مسن، با تأکید بر داروهای سایکوتروپیک، و شیوه مصرف و تجویز دارو در افراد کهنسال مبتلا به اختلالات روانی و نگرانی های مربوط به آن می پردازد.

Title: کمبود آنزیم بیوتینیداز و گزارش ۳ مورد تشخیص داده‌شده آن در بیمارستان مفید

http://rehabilitationj.uswr.ac.ir/article-1-621-fa.html

بیماری کمبود آنزیم بیوتینیداز از بیماری های متابولیک نادر مادرزادی است که اگر در مراحل اولیه زندگی تشخیص داده شود براحتی قابل درمان بوده و از عقب ماندگی ذهنی و جسمی دائم در بیمار پیشگیری خواهد کرد. بیماری به صورت اتوزومال مغلوب به ارث می رسد و شیرخوار دارای علائمی چون اسیدوز لاکتیک، آلوپسی، آتاکسی، پاراپلژی اسپاستیک، تشنج و تأخیر تکاملی است. هم چنین در پوست بیماران تحریکات پوستی اریتماتوز قابل مشاده یوده و اختلال شنوایی و بینایی نیز از دیگر علائم بیماری است. بیمارانی که معرفی می شوند دچار نقص آنزیم بیوتینیداز بودند که در بیمارستان مفید به تشخیص این بیماری رسیده اند. بیمار اول شیرخوار 3ماهه و مذکر و بیمار دوم شیرخوار 5ماهه و مذکر که هر دو با تشنج های مکرر و تأخیر تکاملی مراجعه نمودند. در معاینه متوجه آلوپسی و اسپاستیسیتی اندام ها شدیم و در بررسی های به عمل آمده افزایش لاکتات، آمونیاک و اسیدوز متابولیک مشاهده گردید. در هر دو مورد سطح سرمی آنزیم بیوتینیداز کاهش قابل ملاحظه ای نشان داد که کلیه علائم بیماران با بیوتین برطرف گردید. مورد سوم شیرخوار 8 ماهه و مؤنث بود که با تأخیر تکاملی و لکه های اریتماتوز بدور دهان و مقعد مراجعه نمود که در این مورد نیز پس از تشخیص، با درمان بیوتین کلیه علائم بیمار بهبود یافت.

Title: شیرخواری با اندام‌های بی‌نهایت کوتاه

http://rehabilitationj.uswr.ac.ir/article-1-622-fa.html

سندرم گرب یک دیسپلازی آکرومزوملیک ارثی مغلوب است. در این گزارش یک شیرخوار 5ماهه تهرانی مورد بررسی قرار گرفت. فنوتیپ او به صورت سیستم اسکلتی محوری طبیعی و اندام های بسیار کوتاه و بد شکل بود. اندام های فوقانی و تحتانی بسیار کوتاه بودند .در رادیوگرافی هومروس و فمور نسبتاً قابل رویت بوده و اولنا، فیبولا و تیبیا اصلاً وجود نداشت. قسمتی از رادیوس در رادیوگرافی به صورت دفرمه دیده می شد . مفاصل کارپ، تارس، دست و پا وجود نداشتند و انگشتان به شکل دکمه دیده می شدند.
